# Rehabilitation training combined acupuncture for limb hemiplegia caused by cerebral hemorrhage: A protocol for a systematic review of randomized controlled trial

**DOI:** 10.1097/MD.0000000000014726

**Published:** 2019-03-01

**Authors:** Guang-Fu Song, Cheng-Ji Wu, Shu-Xin Dong, Chang-Hong Yu, Xin Li

**Affiliations:** a Department of Neurosurgery; b Fourth Ward of Neurology Department; c Department of Gastroenterology; d Department of Neurology, First Affiliated Hospital of Jiamusi University, Jiamusi, China.

**Keywords:** acupuncture, cerebral hemorrhage, effectiveness, limb hemiplegia, rehabilitation training, safety, systematic review

## Abstract

**Background:**

Previous studies have reported that rehabilitation training combined acupuncture (RTA) can be used for the treatment of limb hemiplegia (LH) caused by cerebral hemorrhage (CH). However, its effectiveness is still unclear. In this systematic review study, we aim to evaluate the effectiveness and safety of RTA for LH following CH.

**Methods:**

We will retrieve the databases of CENTRAL, EMBASE, MEDILINE, CINAHL, AMED, CBM, and CNKI from inception to March 1, 2019 with no language restrictions. The randomized controlled trials of RTA for evaluating effectiveness and safety in patients with LH following CH will be included. Cochrane risk of bias tool will be used to measure the methodological quality for all included studies. Two authors will independently select the studies, extract the data, and assess the methodological quality of included studies. A third author will be invited to discuss if any disagreements exist between 2 authors. If more than 2 eligible studies will be included, the outcome data will be pooled, and meta-analysis will be conducted if it is possible.

**Results:**

This systematic review will assess the effectiveness and safety of RTA for LH caused by CH. The primary outcome includes limbs function. The secondary outcomes consist of muscle strength, muscle tone, quality of life, and any adverse events.

**Conclusion:**

The findings of this study will summarize the current evidence of RTA for LH caused by CH, and may provide helpful evidence for the clinical treatment.

**Dissemination and ethics:**

The results of this study will be published in peer-reviewed journals or will be presented on conference meeting. This work does not require ethic approval, because it will be conducted based on the published studies.

**Systematic review registration:**

PROSPERO CRD42019120034.

## 1 Introduction

Cerebral hemorrhage (CH) is a very common cerebrovascular disease, which involves rupturing of blood vessels within the brain tissue.^[1–3]^ Previous studies have found that it accounts for 10% to 15% of strokes.^[4,5]^ Additionally, it is also the leading cause of morbidity and mortality in stroke patients,^[6–9]^ with mortality rate of 30% to 50% and about 75% of the survivors cannot live independently after 1 year later.^[4,10]^ The rates of prevalence and incidence are expected to continue rising due to the increasing elderly population.^[11–13]^ Additionally, patients with this condition often complain with problems of motor, sensory, speaking and walking, especially for the limb hemiplegia (LH).^[14–16]^

Although current clinically emergency interventions of lowering blood pressure and surgical craniotomy have been applied very well, many patients still experience LH after the treatment.^[17–19]^ Lots of physical therapies are reported to help LH recovery,^[20–27]^ especially for acupuncture and rehabilitation training.^[20,24,28–40]^ However, no study has systematically evaluated the effectiveness and safety of rehabilitation training plus acupuncture (RTA) for the treatment of LH after CH with higher level evidence. Therefore, this systematic review aims to assess the effectiveness and safety of RTA for the treatment of patients with LH following CH.

## 2 Objective

This systematic review aims to investigate the effectiveness and safety of RTA for the treatment of LH caused by CH.

## 3 Methods

### 3.1 Study registration

This review protocol has been registered in PROSPERO with CRD42019120034. It was developed in accordance with the Preferred Reporting Items for Systematic Reviews and Meta-Analysis Protocol statement guidelines.^[41]^

### 3.2 Participants/population

Patients with LH following CH, regarding sex, age, and race will all be included. However, patients are diagnosed with LH before the CH, or result from other disorders, except the CH will be excluded.

### 3.3 Interventions/exposure

Intervention of any types of rehabilitation training plus RTA will be considered. However, the combination of other intervention types with RTA or rehabilitation training or acupuncture, but not the RTA alone will also be excluded. The control interventions can be any kinds of therapies, except any types of RTA.

### 3.4 Study types

Original studies of randomized controlled trials (RCTs) of RTA for the treatment of LH following CH will be included without any restrictions. However, the studies will be excluded if they are animal studies, reviews, case reports, observational studies, qualitative studies, crossover studies, letters, comments, expert opinions, and duplicated publications.

## 4 Outcome measurements

### 4.1 Primary outcome

The primary outcome includes limbs function, as measured by the Fugl–Meyer Assessment scale, or other associated scales.

### 4.2 Secondary outcomes

The secondary outcomes include muscle strength, as assessed by the motricity index or other related score tools; muscle tone, as evaluated by modified Ashworth scale, or other relevant scales; and quality of life, as examined by activities of daily living scale or any other specific scales. In addition, adverse events are also assessed.

### 4.3 Literature search

The primary source of information will include electronic databases of Cochrane Central Register of Controlled Trials (CENTRAL), EMBASE, MEDLINE, Cumulative Index to Nursing and Allied Health Literature (CINAHL), Allied and Complementary Medicine Database (AMED), Chinese Biomedical Literature Database (CBM), and China National Knowledge Infrastructure (CNKI) from inception to March 1, 2019 with no language restrictions. The RCTs that evaluate the effectiveness and safety of RTA for LH following CH will be included. The search strategy details of CENTRAL database are presented in Table 1. Similar strategies will be applied to the other electronic databases in this study.

**Table 1:**
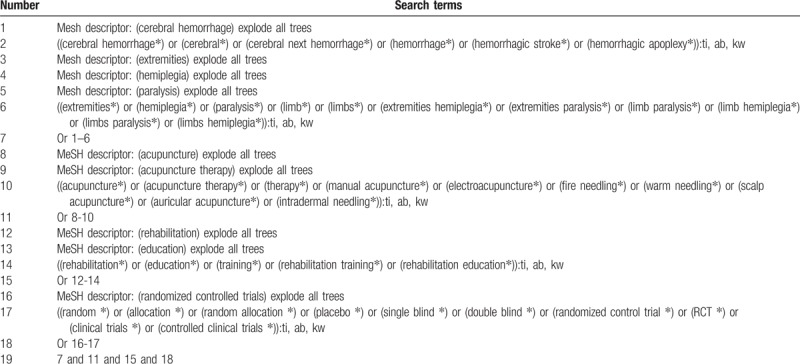
Search strategy applied in CENTRAL database.

The secondary sources consist of Google scholar, website of clinical registrations, reference lists of included studies, and conference proceedings to avoid missing more potential eligible studies.

### 4.4 Study selection

**Figure 1:**
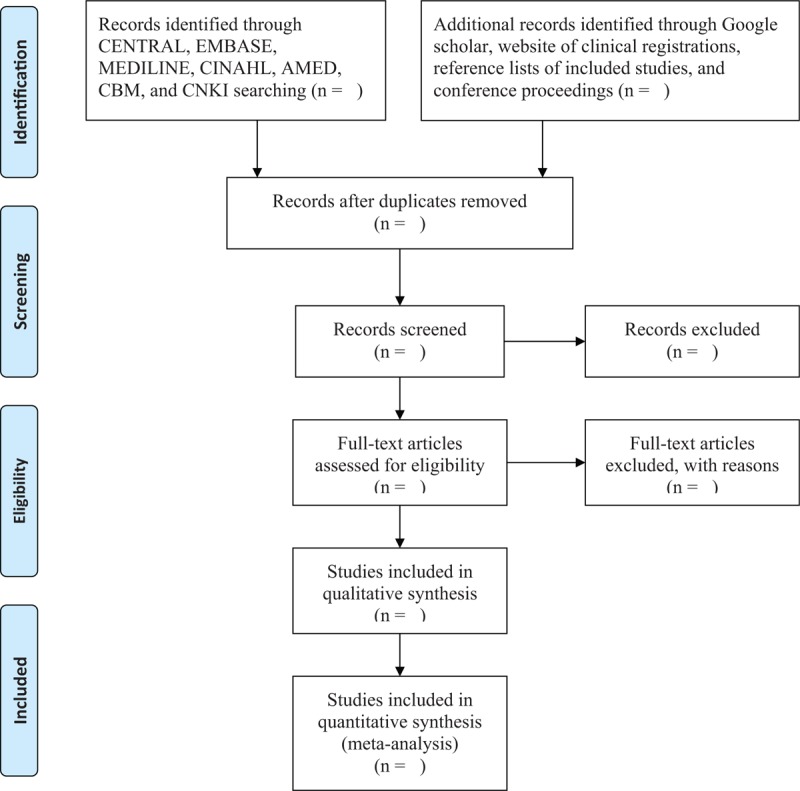
Flow diagram of study selection process.

Two researchers will independently select papers according to the predefined eligibility criteria for screening title or abstract first, and then the full papers will be further read for inclusion if there is insufficient information to judge the study through the title or abstract. The procedures of study selection will be performed in accordance with the PRISMA flowchart and is shown in Fig. 1. Any disagreements regarding the study selection will be dealt with by consulting a third researcher.

### 4.5 Data extraction and management

Two researchers will extract data independently by using a predefined standardized form.

The exacted information consists of general information (authors, year of publication, country, age, sex, ethnicity, disease types, diagnostic criteria, inclusion and exclusion criteria); study design (sample size, details of randomization, allocation, and blinding); details of interventions (dosage, frequency, treatment duration); and outcome measurements (primary and secondary outcomes, adverse events, and any others). Any divergences between 2 researchers will be solved by a third researcher involving through consultation.

### 4.6 Risk of bias evaluation

Two researchers will independently evaluate the methodology quality for all included studies by using Cochrane Handbook for Systematic Reviews of Interventions Tool, which comprises of 7 domains. Each domain will be labeled as low risk of bias, unclear risk of bias, and high risk of bias. Any opposition between 2 authors will be tackled by a third author involved through discussion.

### 4.7 Measurement of treatment effect

In this systematic review, continuous data will be presented as mean difference or standardized mean difference with 95% confidence intervals (CIs). The dichotomous data will be presented as the risk ratio with 95% CIs.

### 4.8 Missing data management

If included study has missing, or insufficient or unclear data, those data will be required from the original authors by contracting them with emails. If those data will not be gettable, this study will only analyze the present available data, and it will be discussed in the text.

### 4.9 Heterogeneity assessment

Tests of *I*^*2*^ and *χ*^*2*^ will be applied to detect the possible heterogeneity. The heterogeneity will be considered as acceptable if the value of *I*^*2*^ is less than 50% (*I*^*2*^ ≤50%). Otherwise, significant heterogeneity will be considered if the value of *I*^*2*^ is more than 50% (*I*^*2*^ >50%).

### 4.10 Data synthesis

We will use RevMan 5.3 software to carry out the data synthesis and meta-analysis. If heterogeneity is acceptable (*I*^*2*^ ≤50%), a fixed-effect model will be utilized to synthesize the data, and meta-analysis will be performed. On the other hand, if heterogeneity is significant (*I*^*2*^ >50%), a random-effect model will be used to pool the data and to operate the meta-analysis. In such a situation, subgroup analysis will be conducted to identify the factors that may cause the significant heterogeneity. If there is still substantial heterogeneity after the subgroup analysis, then data will not be pooled, and meta-analysis will not be conducted. Instead, a narrative summary will be described.

### 4.11 Subgroup analysis

If significant heterogeneity will be detected, subgroup analysis will be carried out in accordance with the different treatments, control interventions, and outcome measurements.

### 4.12 Sensitivity analysis

Sensitivity analysis will be performed to check the robustness of pooled outcome results, methodological quality, and data missing from included trials.

### 4.13 Publication bias

If more than 10 eligible studies will be included, then funnel plot will be performed to detect the publication bias. In addition, the Egg regression and Begger tests will also be conducted to check the funnel plot asymmetry.

## 5 Discussion

The protocol of this systematic review will be conducted to assess the effectiveness and safety of RTA for the treatment of patients with LH following CH. As far as we know, no systematic review and meta-analysis have been addressed to investigate this issue. Therefore, it is very important to conduct this systematic review for assessing the effectiveness and safety of RTA for LH caused by CH.

In the present systematic review, we will search all related studies without language restrictions. All potential studies regarding the effectiveness and safety of RTA for LH following CH will be fully considered. The results of this study may present solid data to future research protocols, and also provide an up-to-date summary of the present evidence on the effectiveness and safety of RTA for patients with LH following CH. The findings of this study may also bring helpful evidence for clinicians.

## Author contributions

**Conceptualization:** Guang-fu Song, Cheng-ji Wu, Xin Li.

**Data curation:** Guang-fu Song, Shu-xin Dong, Chang-hong Yu, Xin Li.

**Formal analysis:** Guang-fu Song, Cheng-ji Wu, Shu-xin Dong, Chang-hong Yu.

**Funding acquisition:** Guang-fu Song.

**Investigation:** Xin Li.

**Methodology:** Guang-fu Song, Cheng-ji Wu, Shu-xin Dong, Chang-hong Yu.

**Project administration:** Xin Li.

**Resources:** Guang-fu Song, Cheng-ji Wu, Shu-xin Dong, Chang-hong Yu.

**Software:** Guang-fu Song, Cheng-ji Wu, Shu-xin Dong, Chang-hong Yu.

**Supervision:** Xin Li.

**Validation:** Cheng-ji Wu, Chang-hong Yu.

**Visualization:** Guang-fu Song, Cheng-ji Wu, Shu-xin Dong, Xin Li.

**Writing - original draft:** Guang-fu Song, Xin Li.

**Writing - review & editing:** Guang-fu Song, Cheng-ji Wu, Shu-xin Dong, Chang-hong Yu, Xin Li.
